# Construct validity and psychosocial correlates of the Italian version of the 21-item Medical Interview Satisfaction Scale in primary care

**DOI:** 10.1192/bjo.2020.164

**Published:** 2021-02-18

**Authors:** Matteo Balestrieri, Giovanni de Girolamo, Paola Rucci

**Affiliations:** Unit of Psychiatry, Department of Medicine, University of Udine, Italy; Unit of Psychiatric Epidemiology and Evaluation, IRCCS Istituto Centro San Giovanni di Dio Fatebenefratelli, Italy; Department of Biomedical and Neuromotor Sciences, Alma Mater Studiorum, University of Bologna, Italy

**Keywords:** Depression, primary care, satisfaction, validity, patient-reported outcomes

## Abstract

**Background:**

Satisfaction with the medical interview has been rarely explored in primary care outside the UK, despite evidence suggesting that a trustful doctor–patient relationship is a key ingredient to facilitate treatment adherence and relief from illness-related distress.

**Aims:**

The aims of this study are to analyse the construct validity of the Italian version of the Medical Interview Satisfaction Scale (MISS-21) and its correlations with two outcome measures, the Inventory of Depressive Symptomatology – Self-Report and World Health Organization Quality Of Life Brief Version, in patients with mild-to-moderate depression, recruited in primary care practices.

**Method:**

The factor structure underlying the MISS-21 was investigated with principal component analysis, and the internal consistency of the factors was evaluated with Cronbach's alpha. Network analysis was used to investigate the interrelationships among items. The importance of individual items in the network structure was determined with centrality analyses. Correlations of MISS-21 scores with changes in depression and quality of life were analysed with Spearman's correlation coefficient.

**Results:**

The MISS-21 proved to have a robust four-dimensional factor structure. Cronbach's alpha for the factors ranged from 0.77 to 0.93, suggesting good to excellent internal consistency. The four factors identified were positively correlated with improvement in depressive symptoms and three quality-of-life domains.

**Conclusions:**

The MISS-21 has sound psychometric properties, and comprises four factors related to clinical outcomes, which makes it suitable for clinical and research applications. The central items in the network should be considered as possible targets for quality improvement interventions in primary care.

## Background

Consistent evidence from mental health research indicates that good treatment outcomes are related to a strong therapeutic alliance between patient and doctor.^1^ The importance of therapeutic alliance and its role in the shared decision-making process have been also demonstrated in non-psychiatric settings.^2^ Alliance depends both on patients’ opinion of their doctor and on the degree of satisfaction with the information received during the medical examination.

Some measures, including the Consultation Satisfaction Questionnaire^3^ and the Patient Satisfaction Questionnaire,^4^ have been developed in the past three decades in UK to assess satisfaction patient with the medical interview, because this assessment was a requirement of the 1990 contract for general practitioners in Britain. Another well-validated instrument is the 21-item Medical Interview Satisfaction Scale (MISS-21), which proved to have good psychometric properties in a UK primary care population.^5^ The MISS-21 was also translated and validated in Malay,^6^ Afrikaans and Xhosa,^7^ and in a modified version (Generic-MISS) in French.^8^ Recently, it has been included among outcome measures in a cluster-randomised trial on depression.^9^ To take into account its worldwide spread and to facilitate comparisons with other studies, we chose to translate and adopt the MISS-21 to assess patients’ satisfaction with the medical interview in a randomised clinical trial on patients with mild-to-moderate depression, carried out in an Italian primary care setting.

## Aims

The aims of this study are first, to analyse the construct validity of the Italian version of the MISS-21; and second, to investigate the correlations of the MISS-21 with the Inventory of Depressive Symptomatology – Self-Report (IDS-SR) and quality of life, measured with the World Health Organization Quality Of Life Brief Version (WHOQOL-BREF) scale.

To accomplish the first aim, we used principal component analysis and network analysis.

Principal component analysis was used to analyse the items structure of the scale, and network analysis was used to investigate the interrelationships among the 21 items.

With reference to the second aim, we hypothesised that patients with a higher satisfaction with the medical interview would be associated with a reduction in depression scores and an increase in the psychological relationships, social relationships and physical domains of the WHOQOL-BREF. Second, we hypothesised that satisfaction with the medical interview would be unrelated with changes in the environment domain of the WHOQOL-BREF.

## Method

### Participants

Data were collected in the framework of a cluster-randomised trial on depression, involving four primary care clinics located in two areas of Northern Italy (Brescia and Udine), for a total of 13 primary care physicians (PCPs).^10^ Two clinics used the experimental protocol, and the other two served as controls, providing treatment as usual.

Inclusion criteria were age between 18 and 65 years and mild-to-moderate depressive symptoms, as measured by Patient Health Questionnaire (PHQ-9) and IDS-SR. In particular, patients had to achieve a score >11 on the PHQ-9 and >26 on the 30-item IDS-SR. Patients who met any of the following diagnostic criteria at baseline were excluded: antidepressant treatment in the previous 3 months, current alcohol or substance dependence, history of bipolar disorder, pregnancy, being in treatment with antipsychotic medications, or any clinical condition requiring in-patient or day hospital treatment.

A complete description of the main study protocol has been reported elsewhere.^10^ In summary, the study compared two different conditions: the telemedicine group and the control group. In the telemedicine group, PCPs had access to an experimental platform and could use all its features. These included support for the PCP in the choice of the best treatment option, thanks to a treatment algorithm, text messaging, availability of psychological support, guidance about the need of a referral to a specialist service and other additional materials of potential help to the PCP. In the control group, PCPs provided treatment as usual, which included all treatment options considered as appropriate by the PCP.

The MISS-21 was translated from English to Italian, and then back-translated to English, by a bilingual person who was blinded to the original version, to ensure semantic equivalence between the two languages.

The 21 items require answers on a 1–7 Likert scale, where 1 denotes strong disagreement and 7 denotes strong agreement with statements concerning specific aspects of individual doctor–patient consultations. The sign of six items (4, 9, 13, 14, 20 and 21) with a negative direction has been inverted for the principal component and network analyses. The Italian version of MISS-21 is provided as Supplemental Appendix 1.

### Instruments and procedures

In addition to the MISS-21, demographic patient characteristics were collected for descriptive purposes and two other questionnaires were administered as patient-reported outcome measures.

The 30-item IDS-SR is designed to assess the severity of depressive symptoms, and includes items that rate the nine symptom criteria used to define a major depressive episode.^11,12^ The overall score on this questionnaire ranges from 0 to 84.^12^

The WHOQOL-BREF measures four domains of quality of life: physical health, psychological relationships, social relationships and environment. It was validated in Italian and proved to have satisfactory psychometric properties.^13^ Domain scores range from 4 to 20.

The IDS-SR and the WHOQOL-BREF were administered at baseline and 6 months, and the MISS-21 was only administered at 6 months.

The authors assert that all procedures contributing to this work comply with the ethical standards of the relevant national and institutional committees on human experimentation and with the Helsinki Declaration of 1975, as revised in 2008. All procedures involving patients were approved by the Comitato Etico Istituzioni Ospedaliere Cattoliche belonging to the IRCCS Istituto Centro San Giovanni di Dio Fatebenefratelli, Brescia (protocol number 21/2013), and by the Comitato Etico Regionale Unico of the Azienda Ospedaliera Universitaria ‘Santa Maria della Misericordia’ di Udine (protocol number 15/2014/Sper). Written informed consent was obtained from all study participants. The trial was registered with Clinicaltrials.gov on 5 October 2012 (identifier NCT01701791).

### Statistical analysis

Continuous variables were summarised with the mean and s.d. or the median and interquartile range, and categorical variables were summarised with absolute and percentage frequencies.

To analyse the structure of the MISS-21 (i.e. to determine whether it measures one or multiple constructs), we carried out a principal component analysis. The number of components (factors) was determined by inspecting the scree plot and selecting the factors with an eigenvalue >1. An oblique (promax) rotation of the correlation matrix was used because factors were correlated. Internal consistency of the scales was measured with Cronbach's alpha. Cut-off values for acceptable, good and excellent internal consistency are ≥0.70, ≥0.80 and ≥0.90, respectively.

A network analysis was then conducted to investigate item correlations, using a graphical model.^14^ The network graphical representation includes nodes (variables) and edges (correlations among variables). Non-parametric correlations were used to take into account the skewness of variables. The least absolute shrinkage and selection operator was used to reduce false-positive edges, and to improve the interpretability of the network. This procedure applies a penalty to small edges by setting them to zero. The shrinkage parameter was chosen to minimise the extended Bayesian information criterion parameter.

The graphical location of nodes was based on the Fruchterman–Reingold algorithm, which places nodes with stronger or more connections close to each other, and nodes with weaker connections at the periphery of the network. Bootstrap analysis with 1000 simulated samples was performed to examine the edge stability.^15^

Three centrality indices of the network were calculated for the 21 variables: betweenness, centrality and degree. The betweenness denotes the number of times a specific node acts as a bridge along the shortest path between two nodes, the closeness measures the number of direct and indirect links between one node and the others, and the degree is the weighted sum of edges (links) of one index node with the other nodes.^15^ Centrality measures were transformed to standardised variables with a mean of 0, and an s.d. of 1 to facilitate comparisons.

To determine whether satisfaction with the medical interview was associated with changes in depression and quality of life, we analysed the correlations of the MISS-21 factor scores with the changes in IDS-SR total score and the WHOQOL-BREF domain scores. Changes in the scale scores were computed as the row difference between baseline and 6 months. To take into account the moderate skewness of these measures, we used Spearman's correlation coefficient. Correlations >0.30 were interpreted as moderate, and those between 0.10 and 0.29 as weak, in line with Cohen's operational definitions.^16^ We hypothesised that a higher satisfaction with the medical interview would be related with lower levels of depression and good quality of life. To address the possible confounding effect of age, gender and treatment protocol on the relationships between MISS-21 factors and changes in depression and quality of file, we carried out generalised multiple linear regression analyses with a gamma distribution function and log link, which do not require that the assumptions of normality and homoscedasticity are met. In these models, we included age, gender and treatment protocol as covariates, and used the ranks of MISS-21 factors and changes in depression and quality of file instead of the original variables.

The JASP package version 0.14.1 for Windows (JASP Team (2020), University of Amsterdam, the Netherlands; see https://jasp-stats.org/download/) was used to perform all statistical analyses.

## Results

The study sample included 64 patients (49 females, 15 males), with a mean age of 48.3 years (s.d. 12.4). No significant differences in the demographic and clinical characteristics were found between patients in the control and the telemedicine group (Table 1), so we carried out the subsequent analyses on the overall sample. The MISS-21 items had a frequency distribution skewed to the left, indicating that most patients reported high satisfaction with the medical interview. (Table 2).

**Table 1:** Characteristics and depression scores at baseline of study participants assigned to the telemedicine group (*n* = 42) and the control group (*n* = 22)

	Overall sample, *n* (%)	Telemedicine group, *n* (%)	Control group, *n* (%)	*χ*^2^ (*P-*value)
Female gender	49 (76.6%)	33 (78.6%)	16 (72.7%)	0.27 (0.60)
Education (≤8 years)	27 (42.2%)	15 (35.7%)	12 (54.5%)	2.10 (0.15)
Employed	21 (32.8%)	15 (37.5%)	6 (27.3%)	0.47 (0.49)
Single	16 (25.0%)	11 (26.1%)	5 (22.7%)	0.09 (0.76)
Past history of depression	27 (42.2%)	19 (45.2%)	8 (36.4%)	0.47 (0.49)
	Mean (s.d.)	Mean (s.d.)	Mean (s.d.)	*t*-test (*P-*value)
Age, years	48.3 (12.4)	47.5 (12.0)	49.7 (11.9)	−0.67 (0.50)
PHQ-9	15.2 (4.0)	15.0 (4.7)	15.6 (2.1)	−0.60 (0.63)
IDS-SR	37.3 (8.8)	37.5 (8.9)	37.0 (8.9)	0.26 (0.80)

PHQ-9, Patient Health Questionnaire; IDS-SR, Inventory of Depressive Symptomatology – Self-Report.

**Table 2:** Descriptive statistics of the 21 items of the Italian version of the Medical Interview Satisfaction Scale

	Mean	s.d.	Median
1.The doctor told me just what my trouble is	5.5	1.7	6.0
2. After talking with the doctor, I know just how serious my illness is	5.2	1.6	5.5
3. The doctor told me all I wanted to know about my illness	5.2	1.8	6.0
4. I am not really certain about how to follow the doctor's advice^a^	2.9	1.7	3.0
5. After talking with the doctor, I have a good idea of how long it will be before I am well again	4.8	1.7	5.0
6. The doctor seemed interested in me as a person	6.1	1.3	6.5
7. The doctor seemed warm and friendly to me	6.2	1.2	7.0
8. The doctor seemed to take my problems seriously	6.1	1.3	6.5
9. I felt embarrassed while talking with the doctor^a^	2.5	1.7	2.0
10. I felt free to talk to this doctor about private matters	5.7	1.7	6.0
11. The doctor gave me a chance to say what was really on my mind	6.0	1.3	6.0
12. I really felt understood by my doctor	5.7	1.2	6.0
13. The doctor did not allow me to say everything I had wanted about my problems^a^	2.7	2.0	2.0
14. The doctor did not really understand my main reason for coming^a^	2.8	1.9	2.0
15. This is a doctor I would trust with my life	5.7	1.5	6.0
16. The doctor seemed to know what (s)he was doing	6.1	1.3	7.0
17. The doctor has relieved my worries about my illness	5.5	1.7	6.0
18. The doctor seemed to know just what to do for my problem	5.6	1.6	6.0
19. I expect that it will be easy for me to follow the doctor's advice	5.3	1.4	5.0
20. It may be difficult for me to do exactly what the doctor told me to do^a^	3.2	1.9	3.0
21. I'm not sure the doctor's treatment will be worth the trouble it will take^a^	2.9	1.7	3.0

a. Item was inverted for the analyses.

The principal component analysis extracted four factors with an eigenvalue >1. Table 3 shows the item loadings to the factors, arranged in decreasing order. The ‘distress relief’ factor included items 1, 2, 3, 5 and 18; the ‘communication comfort’ factor included items 6, 7, 8, 15, 16 and 17; the ‘adherence intent’ factor included items 4, 13, 14, 19, 20 and 21; and the ‘rapport’ factor included items 9, 10, 11 and 12. Items with cross-loadings (17, 21, 14, 19 and 9) were assigned to the factor with the highest loading. The four factors correlated with each other, from 0.177 (adherence intent with rapport) to 0.452 (distress relief with communication comfort). The internal consistency of the factors was good, with Cronbach's alpha ranging from 0.770 to 0.928.

**Table 3:** Results of principal component analysis of the items of the Italian version of the Medical Interview Satisfaction Scale (item loadings are arranged in decreasing order)

	1. Distress relief	2. Communication comfort	3. Adherence intent	4. Rapport
2. After talking with the doctor, I know just how serious my illness is	0.990			
1. The doctor told me just what my trouble is	0.910			
3. The doctor told me all I wanted to know about my illness	0.875			
5. After talking with the doctor, I have a good idea of how long it will be before I am well again	0.840			
18. The doctor seemed to know just what to do for my problem	0.693			
6. The doctor seemed interested in me as a person		0.957		
15. This is a doctor I would trust with my life		0.805		
7. The doctor seemed warm and friendly to me		0.735		
16. The doctor seemed to know what (s)he was doing		0.595		
17. The doctor has relieved my worries about my illness	0.494	0.516		
8. The doctor seemed to take my problems seriously		0.495		
20. It may be difficult for me to do exactly what the doctor told me to do^a^			0.860	
21. I'm not sure the doctor's treatment will be worth the trouble it will take^a^	0.362		0.748	
14. The doctor did not really understand my main reason for coming^a^			0.601	0.351
4. I am not really certain about how to follow the doctor's advice^a^			0.574	
13. The doctor did not allow me to say everything I had wanted about my problems^a^			0.567	
19. I expect that it will be easy for me to follow the doctor's advice	0.307		0.515	
11. The doctor gave me a chance to say what was really on my mind				0.863
10. I felt free to talk to this doctor about private matters				0.854
9. I felt embarrassed while talking with the doctor^a^	−0.449		0.498	0.594
12. I really felt understood by my doctor	0.315			0.569

a. Item was inverted for the analysis.

**Fig. 1:**
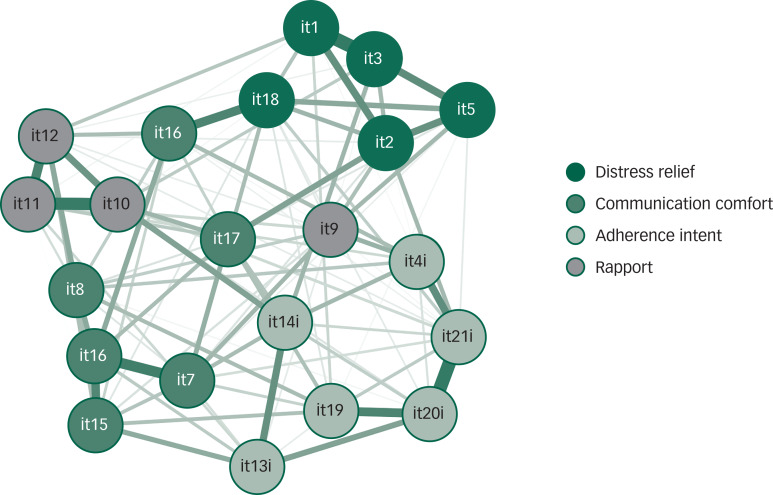
Network structure of the Italian version of the Medical Interview Satisfaction Scale. Items with a suffix ‘i’ have been inverted for the analysis. For the meaning of the items, see Table 2. Edges denoting direct associations are in violet, those denoting inverse associations are in red.

Network analysis was then conducted to examine in further detail the interrelationships among the MISS-21 items, and to identify the most central items. Notably, the items belonging the rapport and distress relief, and communication comfort and adherence intent factors were spatially clustered and strongly interrelated, confirming the robustness of the four constructs identified with principal component analysis (Fig. 1).

**Fig. 2:**
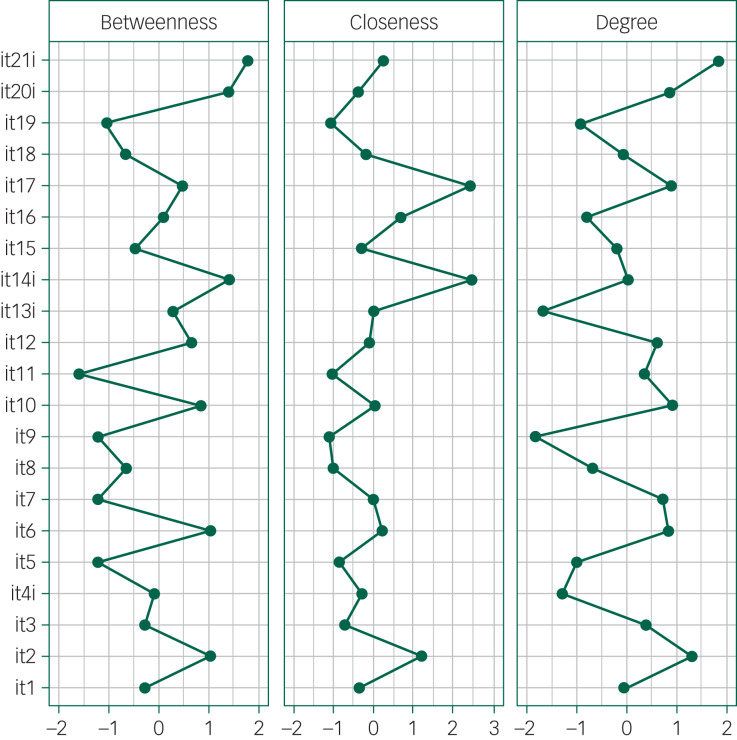
Centrality plot for the Italian version of the Medical Interview Satisfaction Scale network. Betweenness indicates the number of times a node lies on the shortest path length between any two other nodes. Closeness indicates the average distance of a node from all other nodes in the network. Degree quantifies the extent to which a certain node influences other nodes in the network. For each index, higher values reflect greater centrality in the network.

Concerning centrality, examination of Figs. 1 and 2 showed that two items (14, ‘being understood’ and 17, ‘being relieved’) are central to the network because they have many direct and indirect connections with all the other items (high closeness).

Within each factor some items had high centrality indices. Among these, item 2 (‘After talking with the doctor, I know just how serious my illness is’) had the strongest connections with the other items in the distress relief factor. In the communication comfort factor, item 6 (‘The doctor seemed interested in me as a person’) had the highest degree. The adherence intent factor was characterised by more than one item with high centrality: item 21 (‘I'm (not) sure the doctor's treatment will be worth the trouble it will take’) and item 20 (‘It may be difficult for me to do exactly what the doctor told me to do’) had high betweenness and degree, whereas item 14 had both high closeness and betweenness. In the rapport factor, three items were strongly interlinked: item 10 (‘I felt free to talk to this doctor about private matters’), item 11 (‘The doctor gave me a chance to say what was really on my mind’) and item 12 (‘I really felt understood by my doctor’).

**Fig. 3:**
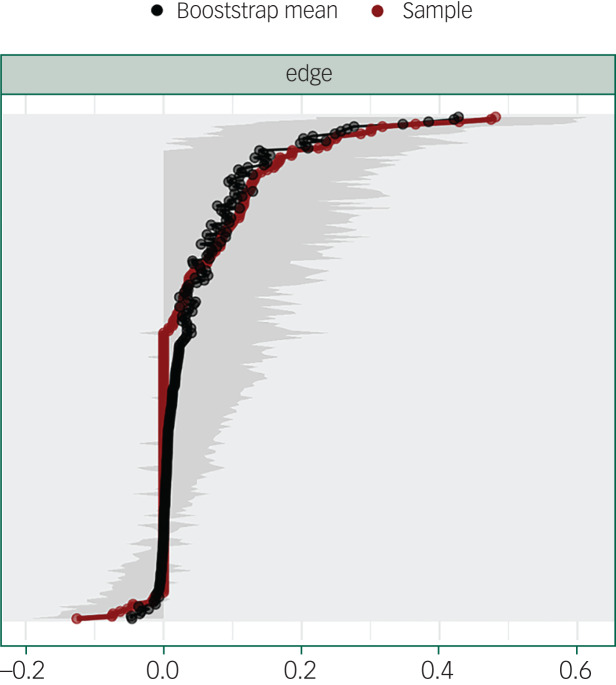
Edge weights (*x*-axis) sorted in increasing order (red line). The black line is the mean of 1000 bootstrap replications. The grey areas are the 95% confidence intervals. In the *y*-axis, the edge labels are omitted to avoid cluttering. The accuracy of edge weights was measured by the mean and 95% confidence intervals of 1000 bootstrap samples drawn from the study population: the narrower the confidence interval, the more accurate is the estimate of the edge weight. The black line in the figure shows that the average weights from the bootstrap samples overlap to large extent with edge weights of the sample (red line). Moreover, the edges with the highest absolute partial correlation are significantly different from those with the lowest absolute partial correlation (confidence intervals do not overlap), but the confidence intervals of many edge weight estimates are quite large. Thus, the edge weight estimations are partly accurate.

Bootstrap analysis indicated that correlations in the network were estimated with an acceptable accuracy (Fig. 3).

We then explored the potential clinical utility of MISS-21, by investigating the correlations between MISS-21 factor scores with the change in depression severity and quality-of-life domains from baseline to 6 months. We found that distress relief had a positive weak correlation with changes in depression severity (ρ = 0.28, *P* = 0.025), positive weak correlations with changes in the psychological and social relationships domains of the WHOQOL-BREF (ρ = 0.268, *P* = 0.034; ρ = 0.290, *P* = 0.022) and a moderate correlation with the physical domain (ρ = 0.316, *P* = 0.012) (Table 4). Communication comfort was only weakly related with changes in depression severity (ρ = 0.272, *P* = 0.030); adherence intent was moderately correlated with changes in the psychological domain (ρ = 0.371, *P* = 0.003), and weakly correlated with the social relationships domain (ρ = 0.286, *P* = 0.024). The rapport factor was unrelated to changes in depression and quality-of-life scores. None of the MISS-21 factors were correlated with the environment domain of the WHOQOL-BREF. After adjusting for gender, age and treatment in multiple regression models, the significant associations between the MISS-21 scores and the outcomes reported in Table 4 were confirmed, except for that of communication comfort with change in depression, which achieved a significance level of 0.065.

**Table 4:** Spearman's correlation coefficients of the MISS-21 factor scores with changes in severity of depression (ΔIDS-SR) and in the quality-of-life domains

	ΔIDS-SR	Δphys	Δpsy	Δrel	Δenv
Distress relief	0.280*	0.316*	0.268*	0.290*	0.087
Communication comfort	0.272*	0.114	0.121	0.204	0.108
Adherence intent	0.226	0.167	0.371**	0.286*	0.219
Rapport	−0.062	−0.031	−0.112	0.001	−0.01

MISS-21, Italian version of the Medical Interview Satisfaction Scale; ΔIDS-SR, change in Inventory of Depressive Symptomatology-Self-Report; Δphys, change in the WHOQOL physical domain; Δpsy, change in the WHOQOL psychological domain; Δrel, change in WHOQOL social relationships domain; Δenv, change in WHOQOL environment domain.

*P < 0.05, **P < 0.01.

## Discussion

The first aim of this study was to investigate the construct validity of the Italian version of MISS-21. The principal component analysis confirmed that the four components of the MISS-21 represent distinct, but correlated constructs.

In the UK version, the MISS-21 was found to consist of four domains: distress relief, communication comfort, rapport and adherence intent.^5^ Although the factors identified in the present study were the same, the items loading to them differed to some extent from the UK version. A possible reason for this discrepancy is that we used a different method to extract the factors (i.e. an oblique rotation of the correlation matrix), which is more appropriate when factors measure interrelated constructs.

In addition, we used network analysis to identify the central aspects in the doctor–patient relationship, which could be used to improve the quality of service provision.^17^ Differently from principal component analysis, network analysis is based on partial item correlations, where each edge represents the relationship between a pair of items after controlling for the correlations with all the other items. Thus, the edge connecting pairs of items reflect their unique associations. Our results show the convergence of the findings from ‘classical’ factor analysis and those from network analysis, corroborating the evidence concerning the multidimensionality of MISS-21 structure.

The network structure indicates that the four factors, although spatially distinct, are interconnected with each other. If we consider the within-factor associations, we can identify some items that play a central role. For instance, the centrality of item 2 (‘After talking with the doctor, I know just how serious my illness is’) in the distress relief factor suggests that it is essential that the doctor gives clear and honest information to the patient. In the communication comfort factor, the centrality of item 6 (‘The doctor seemed interested in me as a person’) underlines the importance for a suffering person to be recognised as a unique person. In the adherence intent factor, the interrelationships and the centrality of items 20 and 21 underscore the key role of patients’ worries about being able to comply with medical indications, which should be addressed by the treating physician. Finally, in the rapport factor, which measures the level of trust in the doctor, three items are strongly interconnected; however, item 9 (which measures patient's embarrassment) has lower correlations with the others, probably because patient uneasiness can have different causes.

Concerning the between-factor associations, the main evidence is that ‘being understood’ and ‘being relieved’ (items 14 and 17), with their elevated closeness, are the core aspects of patients’ satisfaction with the medical interview, and act as a bridge with all of the factors.

As to the correlations between satisfaction with the medical interview and changes in the severity of depression and quality of life, we found that distress relief is associated with both outcomes, whereas adherence intent was moderately correlated with the changes in the psychological and social relationships domains. In other words, our results indicate that lower depressive symptoms and better quality of life at end-point are associated with the ability of the physician to explain the characteristics of the disease, with treatment adherence and with patients’ feeling of being understood by their doctor. Since the study has a cross-sectional design, it is not possible to establish a causal relationship between changes in quality of life, depression and patient satisfaction with the medical interview, because the temporal sequence among these variables cannot be determined. However, the clinical implications of these findings are potentially relevant. If we assume that clinical improvement depends, to some extent, on patient satisfaction, it is important to improve the physician's communication skills. On the other hand, if we assume that improvement in depressive symptoms and quality of life increase the satisfaction with the doctor, we can conclude that the treatment effectiveness has a positive impact on the doctor–patient relationship. This could improve the therapeutic alliance, with positive consequences for future treatments.

In conclusion, our results indicate that MISS-21 has a robust four-dimensional structure, and that receiving clear and honest information, being recognised as a person with a unique identity, expressing concerns about therapy and speaking freely with the doctor are the central aspects of patients’ satisfaction with the interview.

Moreover, satisfaction with the medical interview is associated with positive depression outcomes and better quality of life. These results may lend to different interpretations. In any case, our data confirm that satisfaction with the medical interview is important to promote treatment adherence and therefore improve its effectiveness. MISS-21 may have several potential applications. These include first evaluation of service provision. Low scores on the different components of satisfaction can draw attention on the need to undertake a programme of training in consultation skills to improve relationships with patients. Moreover, the questionnaire offers a multidimensional measure that can be used in research into the determinants of the outcome of care. Studies of patient satisfaction should be used to increase our understanding of patients’ experience about care, and so help make PCP's work more effective.

## Data Availability

The data that support the findings of this study are available from the corresponding author, M.B., upon reasonable request.

## Supplementary material

For supplementary material accompanying this paper visit http://dx.doi.org/10.1192/bjo.2020.164. click here to view supplementary material
