# Supplementary material for: Construct validity and psychosocial correlates of the Italian version of the 21-item Medical Interview Satisfaction Scale in primary care
Source: BJPsych Open. 2021 Feb 18;7(2):e57. doi: 10.1192/bjo.2020.164 (PMC8058927; doi:10.1192/bjo.2020.164)
Supplement: Supplementary file 1 [file S2056472420001647sup001.pdf]

Supplementary Appendix 1

MISS-21 - Medical Interview Satisfaction Scale

Versione Italiana

Balestrieri M., de Girolamo G., Rucci P. (2020)

Si può essere più o meno soddisfatti di una visita medica. Questa scala misura il Suo livello di soddisfazione per l'ultima visita medica che ha effettuato.
Le chiediamo di segnare con una crocetta le caselle che indicano il suo grado di accordo con ciascuna delle 21 frasi contenute nella scala.

	Del tutto in disaccordo	Molto in disaccordo	In disaccordo	Incerto	D' accordo	Molto d' accordo	Del tutto d' accordo
1. Il medico mi ha spiegato quale è esattamente il mio problema	1	2	3	4	5	6	7
2. Dopo aver parlato con il medico, so precisamente quanto è grave la mia malattia	1	2	3	4	5	6	7
3. Il medico mi ha detto tutto quanto volevo sapere circa la mia malattia	1	2	3	4	5	6	7
4. Non sono molto sicuro su come seguire i consigli del medico	7	6	5	4	3	2	1
5. Dopo aver parlato con il medico, ho un'idea chiara di quanto ci vorrà prima di tornare a stare bene	1	2	3	4	5	6	7
6. Il medico sembrava interessato a me come persona	1	2	3	4	5	6	7
7. Il medico mi sembrava disponibile e amichevole	1	2	3	4	5	6	7
8. Il medico sembrava considerare i miei problemi seriamente	1	2	3	4	5	6	7
9. Mi sentivo a disagio mentre parlavo con il medico	7	6	5	4	3	2	1
10. Mi sono sentito libero di parlare con il medico di questioni private	1	2	3	4	5	6	7
11. Il medico mi ha dato la possibilità di dire realmente ciò che avevo in mente	1	2	3	4	5	6	7
12. Mi sono sentito veramente capito dal medico	1	2	3	4	5	6	7
13. Il medico non mi ha permesso di dire tutte le cose che avrei voluto circa i miei problemi	7	6	5	4	3	2	1
14. Il medico non ha proprio compreso la ragione principale per cui sono venuto	7	6	5	4	3	2	1
15. Questo è un medico a cui affiderei la mia vita	1	2	3	4	5	6	7
16. Il medico sembrava sapere ciò che faceva	1	2	3	4	5	6	7
17. Il medico mi ha rassicurato circa la mia malattia	1	2	3	4	5	6	7
18. Il medico sembrava sapere esattamente cosa fare per il mio problema	1	2	3	4	5	6	7
19. Credo che mi sarà facile seguire i consigli del medico	1	2	3	4	5	6	7
20. Può essere difficile per me fare esattamente ciò che il medico mi ha detto di fare	7	6	5	4	3	2	1
21. Non sono sicuro che il trattamento del medico varrà la pena rispetto ai problemi che mi darà	7	6	5	4	3	2	1

Da: Meakin & Weinman, Family Practice, 2002
